# Supplementary material for: HIVprotI: an integrated web based platform for prediction and design of HIV proteins inhibitors
Source: J Cheminform. 2018 Mar 9;10:12. doi: 10.1186/s13321-018-0266-y (PMC5845081; doi:10.1186/s13321-018-0266-y)
Supplement: Supplementary file 2 — Additional file 2. Source code of HIVProtI web server. [file 13321_2018_266_MOESM2_ESM.zip › HIVprotI_Source-code/hivprotI/jsmol/jsv_predict2.htm]

JSpecView/JSME/nmrdb/NIH-Resolver demo


|  |  |
| --- | --- |
|  |  |
|  |  |

This page illustrates how we can use JSME (the JavaScript Molecular Editor)

credits:
JSmol and page development: Bob Hanson
JSME: Peter Ertl, Bruno Bienfait
JSpecView: Robert Lancashire, Bob Hanson
nmrdb: Luc Patiny
NIH Resolver: Markus Sitzmann
Java2Script: Zhou Renjian
